# Ultraporous Amine-Functionalized Organosilicas: Tuning Morphology and Surface Chemistry for Adsorption Applications

**DOI:** 10.3390/molecules30142990

**Published:** 2025-07-16

**Authors:** Marlena Bytniewska, Kacper Latusek, Maria Powęzka, Marcin Kuśmierz, Oliwia Kapusta, Mariusz Barczak

**Affiliations:** Institute of Chemical Sciences, Faculty of Chemistry, Maria Curie-Sklodowska University, Maria Curie-Sklodowska Sq. 3, 20-031 Lublin, Poland

**Keywords:** mesoporous organosilicas, hybrid materials, adsorption, diclofenac sodium, co-condensation, functionalization

## Abstract

Highly porous organosilicas were synthesized via direct co-condensation of two monomers, bis (triethoxysilyl) benzene and aminopropyltriethoxysilane, by adjusting the time between consecutive additions of the monomers and the ageing time of the as-obtained samples. The resulting organosilicas exhibited high porosities, with total pore volumes exceeding 2.2 cm^3^/g. Alongside detailed insights into the morphology, structure, and surface chemistry via a broad spectrum of various instrumental techniques, the obtained ultraporous amine-functionalized organosilicas were tested as adsorbents of diclofenac sodium, chosen here as a model drug. The results revealed remarkable differences in the physicochemical properties and adsorption efficiencies among the obtained samples, confirming that the time gap between the addition of the monomers and ageing time can be used to tune the morphological, structural, and chemical features of the obtained organosilicas and, as a consequence, their sorption efficiencies.

## 1. Introduction and Aim of Work

In recent years, the synthesis and functionalization of mesoporous silica-based materials have attracted considerable attention in various fields, including catalysis [1,2,3,4], separation [5,6,7], environmental remediation [8,9,10], and sensing [11,12,13,14], due to their well-defined pore structure, high surface area, and excellent thermal and chemical stability [15,16]. The huge progress in this area is directly associated with the possibility of easy and controllable surface functionalization with various moieties, which significantly expands the applicability of mesoporous silicas in the above-mentioned applications.

Functionalization may be accomplished in two ways: (i) by post-synthesis grafting of desired groups to the surface of template-free materials [17,18,19] or (ii) by one-pot co-condensation of properly chosen silica monomers [20,21]. Although both protocols have specific peculiarities enable obtaining materials differing in the reactivity, pore accessibility, and distribution of organic groups [22], the co-condensation route seems to be more attractive in terms of providing higher loadings of pendant organic functionalities than post-synthesis grafting (while keeping in mind that too high amounts of functionalized monomer may affect the formation of a mesoporous structure).

Particularly, the incorporation of amine functional groups may significantly enhance their interactions with a plethora of environmental pollutants, including heavy metals, organic compounds, pharmaceuticals, and per- and polyfluoroalkyl compounds [23,24,25,26]. Many studies have demonstrated the effectiveness of amine-functionalized SBA-15 materials in environmental remediation applications. Therefore, methods for introducing amine groups into the porous structure of silicas have reached the highest level of sophistication, both in terms of process optimization (through fine-tuning of synthesis parameters) and in terms of the availability of a wide range of amine-functional monomers, which can be easily grafted to silica surface or co-condensed with tetraethoxysilane (TEOS), both enabling the formation of functionalized mesoporous structure with high content and good accessibility of amine groups. Among a wide range of amine organosilanes, the most widely used are (3-aminopropyl)triethoxysilane (APTES), N-[3-(trimethoxysilyl)propyl]ethylenediamine, N-[3-(trimethoxysilyl)propyl]diethylenetriamine, bis [3-(trimethoxysilyl)propyl]amine, and (3-Aminopropyl)methyldiethoxysilane.

Unfortunately, a drawback associated with the use of those monomers is that during the co-condensation process, the amine groups may interact with poly (ethylene oxide) blocks of the porogenic sacrificial template, Pluronic P123, leading to the partial or even complete destruction of the formed ordered porous structure [27]. Generally, organosilanes bearing basic functional groups, such as APTES, that are readily protonated under acidic conditions exhibit a pronounced disruptive effect on the formation of the SBA-15 mesostructure, even at relatively low concentrations, while organosilanes with not easily protonated groups in acidic pH (e.g., thiol, vinyl) have only a minimal disruptive effect on the mesostructural ordering, even when used at higher concentrations [27]. One possible solution to overcome this problem (at least partially) is the extension of the time interval between the addition of the two monomers., i.e., the structure-directing monomer (usually TEOS) and the functionalizing monomer (e.g., APTES).

Another issue worth discussing is that, despite the wide range of available amine organosilicas that can be used as a functionalizing monomer, the vast majority of studies rely on the same structure-forming monomer, which is tetraethoxysilane (TEOS). In fact, there are only a few studies describing the synthesis of silica-based materials using monomers other than TEOS, such as bissilylated (bridged monomers, like 1,4-bis (triethoxysilyl)benzene (BTESB) or 1,2-bis (triethoxysilyl)ethane (BTESE). For example, Dral et al. investigated the use of various organosilica precursors (including BTESB) in the synthesis of mesoporous materials, discussing their impact on stability [28]. Barczak et al. reported mesoporous organosilicas functionalized simultaneously with both pendant amine groups and ethylene/phenylene bridges (BTESE/BTESB monomers), showing the resulting structures, porosities and adsorption properties of the final materials [29]. Pal et al. studied periodic mesoporous organosilicas embedded with phenylene bridges and amine groups, observing that amine-modified BTESB-based silicas showed high CO_2_ uptake (∼1.8 mmol/g) [30]. Similarly, Sim et al. noticed that BTESB-based silicas grafted with amine groups exhibited high CO_2_ uptake (∼3.0 mmol/g) at a fast adsorption rate, attributing this to the high loading of amine groups but also increased framework hydrophobicity, induced by the high content of the incorporated benzene rings [31]. Benzene-bridged amine-functionalized silicas have also been synthesized and investigated for CO_2_ capture by Erans et al. [32]. Tomina et al. reported spherically shaped BTESB-based organosilica materials functionalized with amine groups for the removal of copper (II), nickel (II), and europium (III) ions from aqueous solutions [33]. Finally, Barczak et al. obtained mesoporous silicas by the co-condensation of two or three silica monomers and by varying the time intervals between the addition of individual monomers, demonstrating the possibility of fine-tuning the porous structure and surface chemistry of the resulting silica materials and, consequently, also the sorption and desorption properties [34].

Unfortunately, apart from the examples cited above (and some other reports not cited here), the literature lacks detailed investigations concerning the synthesis of such BTESB/APTES systems, particularly with regard to the effect of synthesis parameters on their final properties. Therefore, the aim of this work is to demonstrate that it is possible to obtain highly porous periodic mesoporous silicas via co-condensation of two monomers (BTESB and APTES) and the important role of some—often neglected—processing parameters on the final properties of these materials.

## 2. Results and Discussion

**Figure 1:**
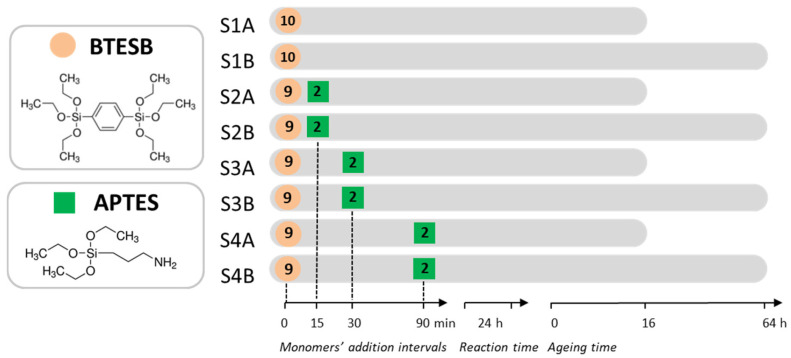
A scheme of the synthesis routes used in this study to obtain the final samples (the numbers inside the circles and squares indicate the amount of BTESB and APTES added in millimoles, respectively).

The sol–gel co-condensation scheme used to obtain amine-functionalized mesoporous silica is schematically shown in Figure 1. To keep the theoretical Si/N molar ratio at 9:1, 9 mmol of bissilylated BTESB was co-condensed with 2 mmol of APTES. The time before adding APTES changed from 15 min (S2A and S2B) to 30 min (S3A and S3B) and finally to 90 min (S4A and S4B).

**Figure 2:**
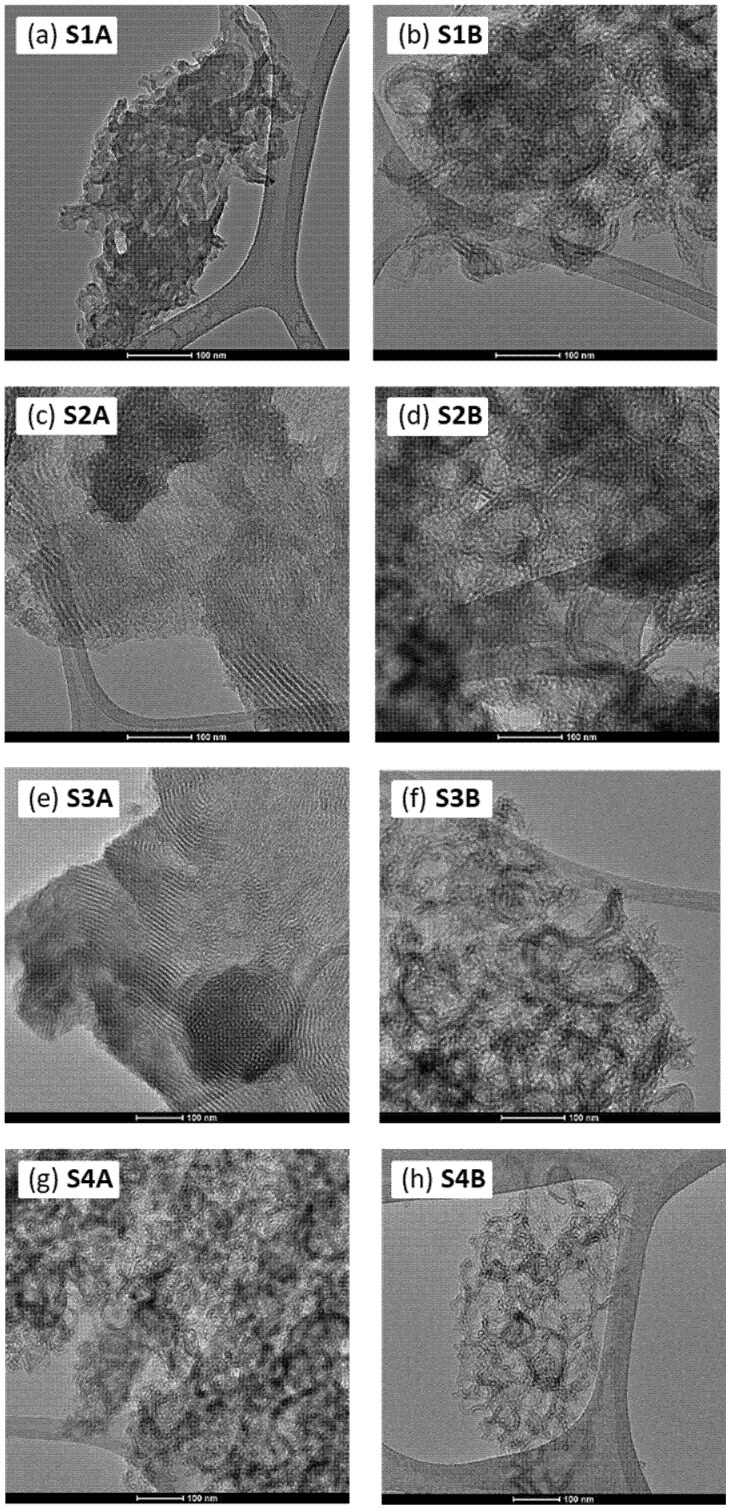
TEM images of the obtained mesoporous silicas (scale bar: 100 nm).

**Figure 3:**
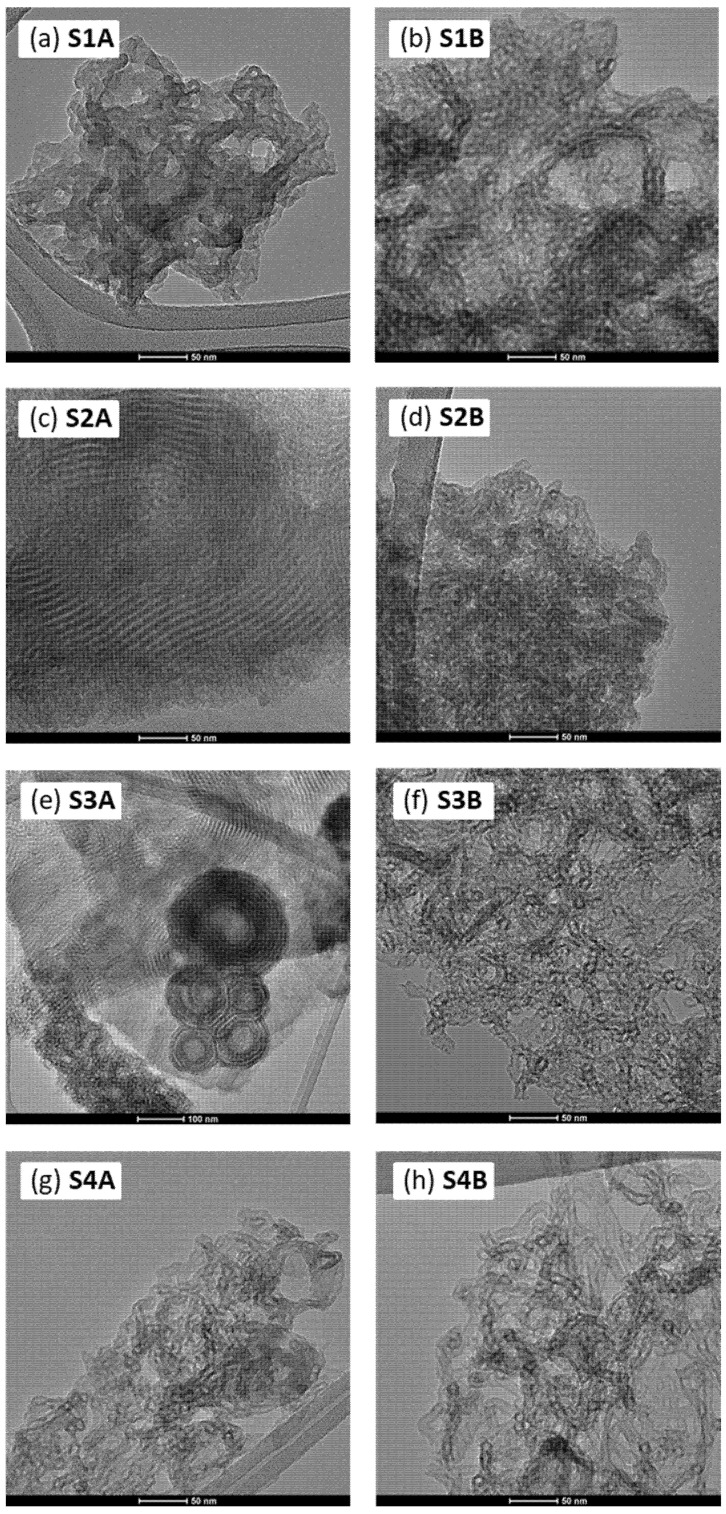
TEM images of the obtained mesoporous silicas (scale bar: 50 nm; for S3A only: 100 nm).

In order to monitor morphological changes resulting from the applied synthesis protocols, transmission electron microscopy (TEM) was used—the selected images are shown in Figure 2 and Figure 3. When comparing the morphology of the obtained BTESB-based samples (S1A and S1B) with typical TEOS-based ones [34], it is seen that the individual cylinders (hexagonal motifs) were often separated from one another, failing to form compact domains of parallelly aligned cylinders. These hexagonal motifs are clearly shorter, and their mutual orientation is of a completely different type than in the case of the silicas, where TEOS was a predominant structure-building monomer [34]. Interestingly, the presence of APTES led to partial densification and ordering of the hexagonal motifs (samples S2A and S3A). However, prolonging the ageing time resulted in final disorganization and the formation of a chaotic structure composed of randomly oriented and highly twisted tubes (samples S2B and S3B). In the case of the samples S4A and S4A, such chaotic morphology is observed regardless of the ageing time applied. The obtained results demonstrated the significant potential that lay in the simple modulation of basic synthesis parameters, such as ageing time. A mere extension of the ageing period could lead to a profound structural reorganization and, consequently, to substantial changes in the properties of the obtained materials (*vide infra*). Interestingly, in some cases, we observed the formation of globular structures (cf. Figure 3e), which appeared to be composed of closed, circular, and parallelly aligned mesopores. The explanation of this intriguing phenomenon went beyond the scope of this brief study and will be the subject of our future research.

**Figure 4:**
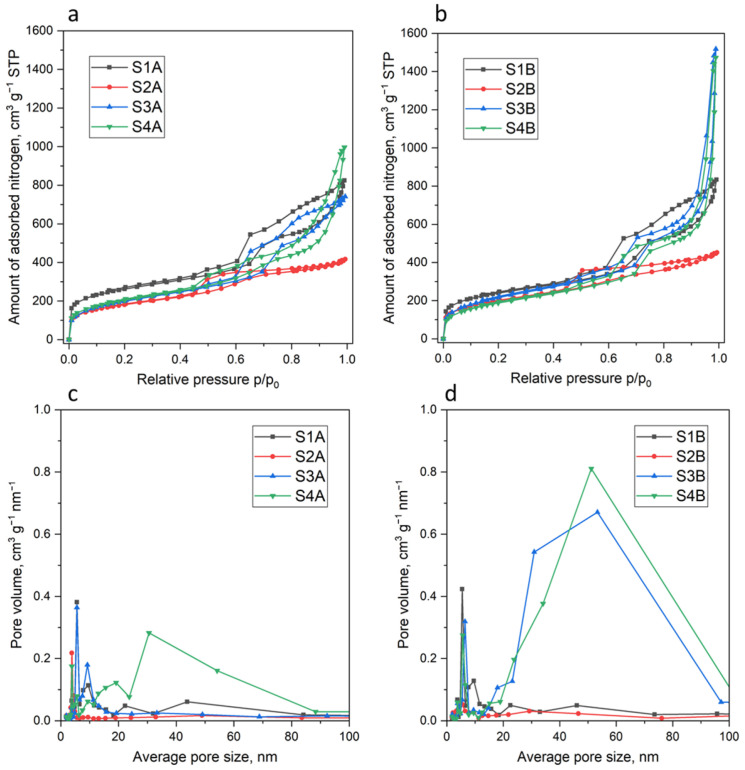
Nitrogen adsorption isotherms of the samples aged for 16 h (**a**) and 64 h (**b**), and the corresponding pore size distributions of the samples aged for 16 h (**c**) and 64 h (**d**).

To acquire deeper insight not only into the morphology but also into the formed porous structure, the obtained materials were further characterized using low-temperature nitrogen adsorption–desorption—the resulting isotherms are presented in Figure 4a,b, the pore size distributions are shown in Figure 4c,d, and the corresponding textural parameters are summarized in Table 1.

All the isotherms are type IV according to the IUPAC classification [35], which is characteristic of mesoporous materials. All isotherms exhibit a hysteresis loop, which is characteristic of mesoporous materials; however, the shapes and positions of these loops on the relative pressure axis vary depending on the sample. It is evident that ageing time has a significant effect on the porosity of the resulting materials; particularly, comparing the samples S3A and S4A (aged 16 h) with their counterparts aged four times longer (S3B and S4B, respectively), it can be seen that prolonged ageing leads to the development of mesoporosity, as indicated by a markedly larger hysteresis loop.

Interestingly, in the case of the samples S3B and S4B, there were two well-separated hysteresis loops (cf. Figure 4b): the first loop was located in the *p*/*p*_0_ range of ~0.6–0.75 and was attributed to the presence of hexagonally arranged mesopores, while the second loop was located in the range ~0.9–1.0, which could be attributed to the aggregates of particles and networks consisting of interparticle mesopores and macropores that are not completely filled with nitrogen [36]. When comparing these data with the TEM images of samples S3B and S4B (where a loose, three-dimensional network composed of randomly oriented nanotubes was clearly seen, cf. Figure 2f–h), it became evident that the increased porosity originated from interspaces between loosely arranged nanotubes.

All the obtained organosilicas had high specific surface areas, S_BET_, in the range of 639–908 m^2^/g (cf. Table 1). The attribution of the surface area to micropores (i.e., smaller than 2 nm) and the remaining pores (i.e., bigger than 2 nm) showed that samples obtained solely through the condensation of BTESB exhibited significantly higher microporosity compared to all other samples. Moreover, the microporosity of the samples S3A, S3B, S4A, and S4B was noticeably lower.

Pore volumes for basic S1A and S1B samples were very close (1.28 and 1.29 g/cm^3^, respectively, cf. Table 1). For samples S2A and S2B, obtained through the relatively rapid addition of APTES (15 min. after the BTESB), a substantial, nearly twofold decrease in pore volume was observed, also accompanied by substantial S_BET_ decline. So, too early addition of APTES may have a detrimental effect on the development of the porous structure. For the samples S3A and S4A, the pore volumes were similar to those for S1A sample, but the prolonged ageing time from 16 h to 64 resulted in a tremendous increase in pore volumes for the samples S3B and S4B as high as 2.35 and 2.28 cm^3^/g, respectively, making the obtained materials among the most porous mesoporous silicas reported in the literature [34,37,38]. Usually, to achieve such developed porosities, tailored expansion strategies are required (e.g., the use of swelling agents), as in the case of the synthesis of highly porous mesocellular foams [39,40]. The pore size distributions presented in Figure 4c,d confirmed the mesoporous nature of the obtained materials, providing also a visual representation of the porosity changes occurring in the studied materials, particularly during prolonged ageing. For example, when comparing samples S3A vs. S3B or S4A vs. S4B, it is clearly visible that the porous structure reoriented toward the development of larger pores, mainly mesopores (possibly also macropores) as a result of the development of extended interparticle pores and intraparticle pores, formed in the case of all the samples.

**Figure 5:**
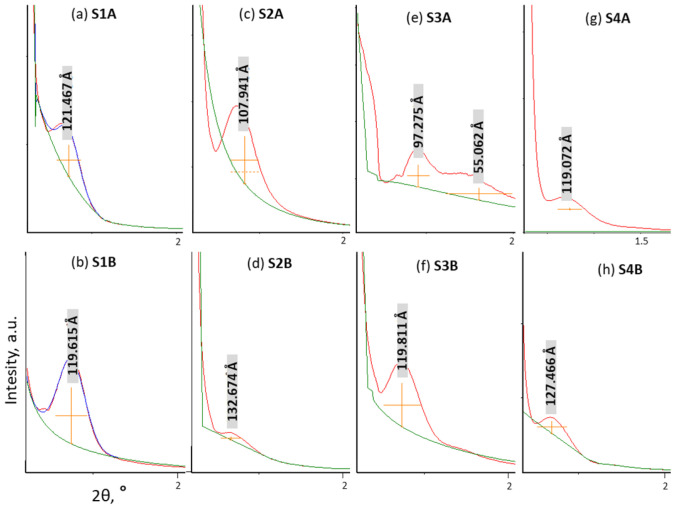
XRD low-angle diffractograms for the studied silicas.

The XRD diffractograms presented in Figure 5 confirmed the above-mentioned findings, showing a poor mesostructural ordering for all the samples, which was manifested by a broad diffraction peak with a maximum of 2θ ≈ 0.7°, consistent with our previous results [34].

**Figure 6:**
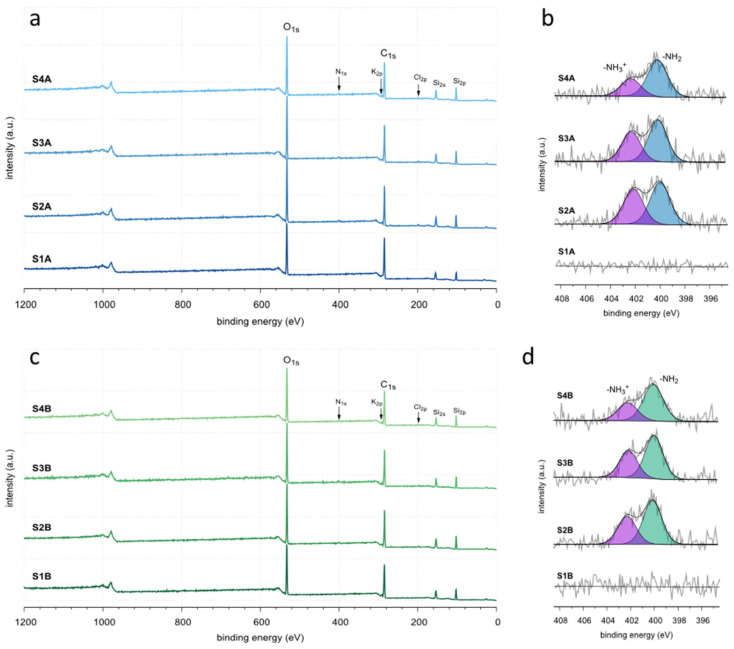
XPS survey spectra for the samples: aged for 16 h (**a**) and for 64 h (**c**), along with deconvoluted N 1 s signals for the samples aged for 16 h (**b**) and for 64 h (**d**).

In addition to changes in morphology, porosity, and structural ordering, the assessment of possible alterations in chemical composition, particularly in surface chemistry, are of great importance, as these may result from variations in synthesis conditions (namely, APTES addition time delay: 15 vs. 30 vs. 90 min, and time of ageing: 16 h vs. 64 h, cf. Figure 1). Therefore, all obtained silicas were characterized using two techniques: CHN elemental analysis, which determines the carbon, hydrogen, and nitrogen content in the bulk phase, and X-ray photoelectron spectroscopy (XPS), which provides information on the surface chemical composition. The results were summarized in Table 1, and the XPS results are also shown in Figure 6.

The results of the elemental analysis showed similar carbon content for all the samples (36.7–39.3 wt.%) and similar hydrogen content (4.6–5.7 wt.%). However, the most important results concern the observed nitrogen content, which reflects the efficiency of the co-condensation between BTESB and APTES. As expected, no nitrogen was detected in samples S1A and S1B, since APTES was not used in their synthesis. For all the remaining samples, the nitrogen content was below 1 wt.%, but in samples S4A and S4B, the nitrogen content was approximately 20% lower than in samples S2A, S2B, S3A, and S3B (cf. Table 1). Presumably, the excessively prolonged delay in APTES addition resulted in reduced BTESB-APTES co-condensation efficiency as a robust and well cross-linked structure was formed due to BTESB condensation; therefore, APTES could not be so successfully co-condensed into the already formed organosilica framework. When comparing pairs of samples, A and B (e.g., S2A and S2B), it is clearly seen that ageing time did not have a significant effect on the observed nitrogen content. This was somewhat expected, as the processes affecting APTES co-condensation occurred during the earlier stages prior to the ageing (i.e., the monomer addition and the subsequent 24 h reaction period, cf. Figure 1).

XPS surface elemental analysis confirmed that in all the cases, carbon was the main component, and its amount varied from 51.2 to 57.9 at.%. Oxygen was the second most abundant element, with amounts ranging from 25.7 to 26.7 at.%, while silica concentrations ranged from 14.9 to 19.4 at.%. Again, the most significant results pertained to the nitrogen content, as it directly reflected the efficiency of the co-condensation between BTESB and APTES. XPS revealed a clear trend here: As the delay in APTES addition relative to BTESB increased, the nitrogen content in the final samples decreased, unequivocally indicating that prolonged delays of APTES addition negatively affected co-condensation efficiency. As mentioned above, this effect was mainly attributed to limited access of not fully hydrolyzed APTES to already hydrolyzed and partially co-condensed BTESB organosilica network. The deconvolution of the N 1 s signal further revealed that a significant fraction of amine groups exist in the protonated form. This may have enhanced the interactions between these protonated groups and the poly (ethylene oxide) chains of P123 template via hydrogen bond formation between the protonated amino groups (–NH_3_^+^) and the free electron pairs present in PEO oxygen atoms.

**Figure 7:**
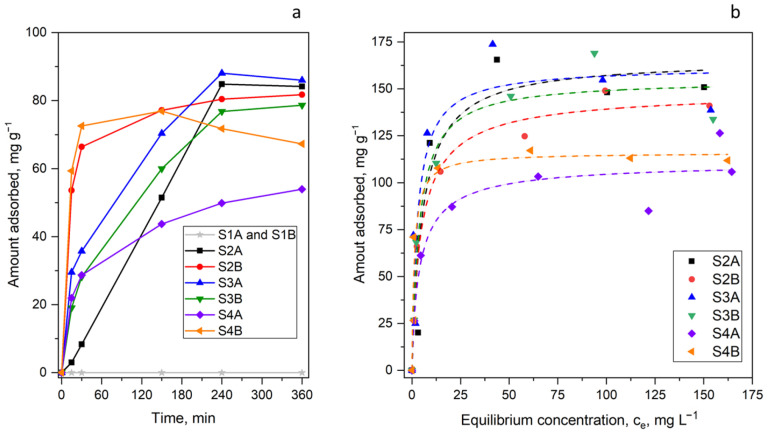
Kinetics of adsorption (**a**) and adsorption isotherms fitted with the Sips (Langmuir–Freundlich) model (**b**).

To demonstrate the possible applications, the obtained organosilicas were tested in a model application: removal of a model drug (diclofenac sodium, DICL) from aqueous solutions. In our previous studies, we showed that DICL adsorption is basically quite resistant to pH changes; thus, adsorption experiments were run in unbuffered water with (pH ≈ 5.5–6.0), as in our previous studies [22,34,41]. Since the pKa value of DICL is 4.15 [41], working within this pH range ensured that the soluble ionized form of DICL was predominantly present in the solution. The adsorption kinetic curves and isotherms are shown in Figure 7, and the observed DICL uptakes (referred to here as static sorption capacities, SSC) along with fitting parameters obtained using the Sips (Langmuir–Freundlich) model are presented in Table 2.

When analyzing the adsorption rates of diclofenac by the individual samples, significant differences between them become apparent. Some samples, such as S2B and S4B, exhibited very rapid adsorption, occurring even within the first 15 min. In contrast, others, such as S2A and S3B, showed a much slower adsorption rate. Organosilicas synthesized without the use of APTES (i.e., S1A and S1B) did not adsorb diclofenac at all, indicating that amine groups played a crucial role in governing the sorption behaviour of all the remaining sorbents. The essential role of amine groups was further confirmed by fitted adsorption isotherms, which showed that samples containing a lower nitrogen content (S4A and S4B, as evidenced by XPS, cf. Table 1) exhibited lower adsorption capacities towards DICL compared to samples with higher nitrogen content (S2A, S2B, S3A, and S3B, cf. Table 1). A quasi-linear relationship (R^2^ = 0.8) could even be observed between the nitrogen surface content (given by XPS, cf. Table 1) and sorption capacity (predicted by the Sips model, cf. Table 2). Naturally, the considerable uncertainties associated with quantifying such low nitrogen levels via XPS on one hand, and determining the sorption capacity from the Sips isotherm on the other, hindered the establishment of an exact correlation. It is also important to note that different fractions of amino groups in various materials exist in either the protonated or unprotonated form. As we have clearly demonstrated in our previous studies, the protonation of amine groups plays a key role in their interactions with the diclofenac anions [41]. This is because the adsorption mechanism of DICL is mainly based on hydrogen bonding between the DICL carboxylate anions and the protonated amine groups, along with electrostatic interactions between these anions and the positively charged silica surface at pH 5.5–6.0.

## 3. Materials and Methods

### 3.1. Reagents

The following reagents were used: 1,4-bis (triethoxysilyl)benzene (BTESB, 96%, Sigma-Aldrich, St. Louis, MO, USA), 3-aminopropyltriethoxysilane (APTS, 98%, Fluorochem, Glossop, UK), Pluronic P123 (P123, Sigma, St. Louis, MO, USA), hydrochloric acid (HCl, 36%, POCH, Gliwice, Poland), sodium hydroxide (NaOH, POCH, Gliwice, Poland), ethanol (EtOH, 99.8%, POCH, Gliwice, Poland), and diclofenac sodium salt (DICL, >98%, Sigma-Aldrich, St. Louis, MO, USA). All chemicals were used as received, without further purification.

### 3.2. Synthesis of the Samples

The general synthesis protocol of the organosilicas described in this study followed our previous works [21,42,43]. The important difference was that, instead of TEOS, we used BTESB as a structure-forming agent [34]. To obtain an amine-free sample (**S1**), first, 2 g of P123 was dissolved in 72 mL of 1.75 M HCl while stirring overnight at 40 °C to achieve the complete P123 dissolution. Then 10 mmol of BTESB was added dropwise. The mixture was stirred for the next 24 h (at 40 °C) and then aged at 100 °C for the next 16 h (sample S1A) or 64 h (sample S1B) to investigate the possible changes due to two different ageing times. The precipitated solids were washed with 40 mL of deionized water, filtered, and dried at 70 °C. The template was removed by triple extraction with acidified EtOH (each portion was composed of 147 mL of 99.8% EtOH and 3 mL of 37% HCl) at an elevated temperature. Then the powders were filtered and washed with pure EtOH and dried at 70 °C.

The amine functionalized samples were obtained analogously but with one significant difference: instead of using 10 mmol of BTESB (as in the case of S1), 9 mmol of BTESB and 2 mmol of APTES were used, and APTES was added 15, 30, and 90 min after the addition of BTESB (S2, S3, and S4, respectively) to investigate whether the time interval between the addition of both monomers would affect the samples’ properties. Again, the samples were aged for 16 h and 64 h, which ultimately resulted in six samples (S1A, S1B, S2A, S2B, S3A, and S3B, respectively).

### 3.3. Instrumental Characterization

The nitrogen isotherms were measured at −196 °C by a 1200e analyzer (Quantachrome Instruments, Boynton Beach, FL, USA). Before analysis, samples were degassed overnight at 110 °C in a vacuum. The BET specific surface areas (S_BET_) were evaluated in the range of relative pressures *p*/*p*_o_ = 0.05–0.20. The total pore volumes (V_t_) were calculated by converting the amount adsorbed at *p*/*p*_o_ ~ 0.99 to the volume of liquid adsorbate. The pore size distributions (PSD) were calculated from the desorption branch of isotherms using the Barrett–Joyner–Halenda (BJH) method. The TEM images were collected using a Tecnai G20 X-Twin (FEI) microscope (FEI, Hillsboro, OR, USA). The CHN elemental analysis was carried out using the Perkin Elmer CHN 2400 analyzer (Perkin-Elmer, Waltham, MA, USA). Powder X-ray diffraction (XRD) patterns were recorded by using an Empyrean X-ray diffractometer (PANalytical, Tokyo, Japan) (CuKα radiation) with a 0.02° size step and a 10 s time step, covering a range of 0.5° < 2θ < 5.0°. X-ray photoelectron spectroscopy (XPS) spectra were collected using the multichamber UHV system (Prevac) and a hemispherical Scienta R4000 electron analyzer (Scienta, Uppsala, Sweden). A Scienta SAX-100 X-ray source (Al Kα, 1486.6 eV, 0.8 eV band) with an XM 650 X-Ray Monochromator (0.2 eV band) was used (Scienta, Uppsala, Sweden). Peak deconvolutions were performed using MultiPak software 6.5.

### 3.4. Sorption and Release of Diclofenac

In each experiment, ~10 mg of adsorbent was shaken for 24 h with 30 mL of DICL solution at a selected concentration. For kinetics measurements, contact times varied between 2 min and 24 h. The equilibrium adsorption amounts were calculated from the following formula: a = (c_i_ − c_j_)·V·m^−1^, where c_i_ is the initial concentration (mg L^−1^, c_j_—the final concentration (mg L^−1^), V—the volume of the solution (L) and m—the mass of the adsorbent (g)). Spectrophotometric determination of DICL concentrations was accomplished using the UV-VIS spectrometer Specord 200 (Analytic Jena, Jena, Germany) at λ_max_ = 278 nm from the filtered solution (0.45 µm syringe filter) to avoid interference from suspended silica particles. The adsorption experiments were conducted at 20 °C ± 1 °C in unbuffered solutions with a pH ≈ 6.

## 4. Conclusions

In this study, we systematically investigated the influence of two usually neglected synthesis parameters (a delay in the addition of the second monomer and the time of ageing) on the structural, morphological, chemical, and sorption properties of amine-functionalized mesoporous organosilicas. The co-condensation of BTESB with APTES enabled us to prepare a series of materials with tunable porosity, surface chemistry, and sorption behaviour. The TEM and nitrogen sorption data revealed that both mesostructural ordering and porosity were highly sensitive to variations in the above-mentioned parameters. Notably, prolonged ageing (64 h) led to the formation of ultraporous materials with specific surface areas exceeding 900 m^2^/g and total pore volumes as high as 2.35 cm^3^/g—values that have been rarely achieved without the use of dedicated pore-expansion procedures. Amine groups’ incorporation efficiency decreased with increasing delay in APTES addition, while it remained unaffected by the prolonged ageing time. The content of amine groups was found to be critical for the adsorption of the chosen model drug (diclofenac sodium, DICL): samples with higher nitrogen content exhibited significantly greater sorption capacities.

The obtained results highlighted the nuanced interplay between synthesis parameters and the resulting properties, simultaneously demonstrating the strategy of selecting these parameters to obtain ultraporous silica-based materials with potential applications in adsorption processes. The demonstrated ability to modulate sorption behaviour through controlled synthesis may be used to facilitate the design and control of both existing and novel sorbents for various applications.

## Figures and Tables

**Table 1:** Selected structural and chemical properties of the studied samples.

Sample	Porous Structure Parameters				CHN Elemental Analysis (wt.%)			XPS Elemental Analysis (at.%)			
	S_BET_ (m^2^/g)	S_ext_ (m^2^/g)	S_mic_ (m^2^/g)	V_t_ (cm^3^/g)	C	H	N	Si	C	O	N
S1A	908	664	244	1.28	37.76	4.58	---	14.9	57.9	26.4	---
S1B	834	631	203	1.29	39.34	5.30	---	18.9	53.5	26.7	---
S2A	639	545	95	0.64	37.64	5.40	0.97	18.8	51.2	26.2	2.1
S2B	700	605	95	0.70	36.67	5.31	0.97	19.4	51.2	25.8	2.1
S3A	717	691	25	1.15	38.75	5.41	0.96	18.6	51.9	26.3	1.8
S3B	785	772	13	2.35	38.87	5.69	0.95	18.4	52.8	25.7	1.6
S4A	727	649	79	1.54	38.82	5.56	0.76	18.5	53.1	26.1	1.1
S4B	682	670	12	2.28	38.87	5.60	0.82	18.0	53.3	26.4	1.1

S_BET_—specific surface area by BET method, S_ext_—specific surface area of pores other than micropores, S_mic_—specific surface area of micropores from t-plot, V_t_—total volume of the pores.

**Table 2:** The Sips (Langmuir–Freundlich) fitting parameters and the observed uptakes (SSC) of the studied organosilicas.

Sample	Sips Fitting				SSC (mg/g)
	q_m_	K_L_	*n*	R^2^	
S1A	---	---	---	---	0
S1B	---	---	---	---	0
S2A	166	0.185	1.000	0.886	166
S2B	150	0.256	0.861	0.980	149
S3A	162	0.355	0.970	0.858	155
S3B	155	0.259	1.000	0.959	168
S4A	111	0.290	0.855	0.935	126
S4B	116	0.805	0.988	0.917	117

## Data Availability

The data presented in this study are available on request from the corresponding author.
